# Changes in home care clients’ characteristics and home care in five European countries from 2001 to 2014: comparison based on InterRAI - Home Care data

**DOI:** 10.1186/s12913-021-07197-3

**Published:** 2021-10-29

**Authors:** I. V. Kristinsdottir, P. V. Jonsson, I. Hjaltadottir, K. Bjornsdottir

**Affiliations:** 1 Faculty of Nursing, University of Iceland, Eiríksgata 34, 101 Reykjavík, Iceland; 2 Home Care center, The Capital Area Primary Care, Álfabakki 16, 109 Reykjavík, Iceland; 3 Faculty of Medicine, University of Iceland, Vatnsmýrarvegur 16, 101 Reykjavík, Iceland; 4 Department of Geriatrics, The National University Hospital of Iceland, Túngata 26, 101 Reykjavík, Iceland

**Keywords:** Home care, Formal care, Elderly, Health care policy, interRAI-home care, IBenC, AdHOC

## Abstract

**Background:**

Policymakers advocate extended residence in private homes as people age, rather than relocation to long-term care facilities. Consequently, it is expected that older people living in their own homes will be frailer and have more complex health problems over time. Therefore, community care for aging people is becoming increasingly important to facilitate prevention of decline in physical and cognitive abilities and unnecessary hospital admission and transfer to a nursing home. The aim of this study was to examine changes in the characteristic of home care clients and home care provided in five European countries between 2001 and 2014 and to explore whether home care clients who are most in need of care receive the care required.

**Methods:**

This descriptive study used data from two European research projects, Aged in Home Care (AdHOC; 2001–2002) and Identifying best practices for care-dependent elderly by Benchmarking Costs and outcomes of Community Care (IBenC; 2014–2016). In both projects, the InterRAI-Home Care assessment tool was used to assess a random sample of home care clients 65 years and older in five European countries. These data facilitate a comparison of physical and cognitive health and the provided home care between countries and study periods.

**Results:**

In most participating countries, both cognitive (measured on the Cognitive Performance Scale) and functional ability (measured on the Activities of Daily Living Hierarchy scale) of home care clients deteriorated over a 10-year period. Home care provided increased between the studies. Home care clients who scored high on the physical and cognitive scales also received home care for a significantly higher duration than those who scored low.

**Conclusion:**

Older people in several European countries remain living in their own homes despite deteriorating physical and cognitive skills. Home care services to this group have increased. This indicates that the government policy of long-term residence at own home among older people, even in increased frailty, has been realised.

## Background

Countries in the Western world have been preparing for demographic aging. In Europe, the proportion of 65 years and older is expected to increase from 16% in 2010 to 27.8% in 2050 [1]. Policymakers in most countries advocate extended residence in private homes as people age, rather than relocation to long-term care facilities. Therefore, community care for aging people is becoming increasingly important as it facilitates postponement of transfer to a nursing home and prevention of unnecessary hospital admissions [2]. This policy has a number of benefits for older people. They have more control over their own lives, and they enjoy increased independence and autonomy. In many cases, older people have established relationships and roles in their community or neighbourhood, and this contributes to their improved well-being. Staying at home also reduces additional health burdens, compared to nursing homes, such as increased infections and antibiotic resistance [3, 4]. Although the elderly may benefit from continued residence at home, such an arrangement also has some drawbacks. Some older people may have unsuitable housing or may feel isolated, while some may need increased assistance or home care services due to their deteriorating health [4].

In many countries, health and social care authorities have developed multifaceted community-based home care services to support older people living independently. This has led to enhanced interest in home care services for the growing population of frail older people living in their own homes. Home care services have been designed to provide specialized treatments and assist with everyday activities that individuals can no longer perform owing to general physical or cognitive impairment, illness, or lack of knowledge and skills. Home care nurses play an important role in such services as they manage long-term health conditions, prevent unnecessary admission to hospitals, and provide clinical leadership in home care [5, 6]. This trend has necessitated improved knowledge and competence among home care staff to address more complex and extensive care needs [7].

In this study, the impact of European health care policy [8] on older people living longer in the community with higher dependency levels was explored. We aimed to investigate whether older people with complex disabilities and care needs remain living in their own homes with the support of home care services. We were also interested in knowing how the services were distributed, i.e., if those in most need received most services. Using data from two studies undertaken in five European countries with a ten-year interval, it was possible to compare older people’s status and circumstances, that is, whether they were living independently and receiving formal and informal care. Formal care refers to paid care provided by healthcare and social institutions, whereas informal care refers to unpaid care provided by family, friends, or neighbours [9]. The older people’s demographic characteristics, health status, skills, conditions, and needs as well as the assistance they received at the two respective times was examined. We also explored cross-national differences so that countries can learn from each other and adopt new methods to provide home care services. In this study, the focus was on home care; however, it is known that the relationship between formal and informal assistance is often indistinguishable.

The present study was based on findings from two European studies, the Aged in Home Care (AdHOC) project study from 2001 [10] and the Identifying best practices for care-dependent elderly by Benchmarking Costs and outcomes of Community Care (IBenC) study from 2014 [11]. In these studies, home care or community care is defined as ‘care provided at home by social and healthcare professionals’ and care as ‘domestic aid services; personal care; and supportive, technical, and rehabilitative nursing’ [12]. In both studies, home care clients were assessed using the interResident Assessment Instrument for home care (interRAI-HC), which is a comprehensive tool designed to provide holistic information on the status of older persons.

The development of the interRAI began by a team of gerontologists in the United States in the late 1980s, following an audit of the activities in nursing homes where the quality of care had been identified as substantially lacking, activities deficient, and their supervision limited [13]. The aim was to develop a tool to obtain a systematic and comprehensive assessment of the health of nursing home residents, which could then be used to monitor changes. The instrument is in two parts: a data set (minimum data set [MDS]) that contains the components of a comprehensive assessment of the older person and outcomes from various scales embedded in the assessment tool such as the client assessment protocols (CAPs), quality indicators, and resource utilization groups (RUGs; which estimates the cost of care). The results provide treatment guidelines and address the main health problems and participants’ general condition, indicating what problems exist or may develop. It was anticipated that the results of such an assessment could be used to develop quality standards for institutions.

The interRAI Nursing Home assessment tool was first implemented in nursing homes in the U.S. in the early 1990s [14]. In the following years, new versions of the assessment tool were developed that were related to different sectors within the health service. In 1996, the interRAI Home Care instrument was introduced for use in assessing home care clients. In addition to assessing cognitive and physical skills, the instrument places emphasis on factors related to quality of life and activity of individuals as well as assistance provided by family caregivers [15, 16].

Since the interRAI-HC instrument was used both in the AdHOC and IBenC studies comparison was made possible. Six European countries participated in the IBenC study (2014–2016): Belgium, Finland, Germany, Italy, Iceland, and the Netherlands. In all these countries, long-term residence in private homes has been encouraged in parallel with increasing age. Five of these countries participated both in the AdHOC study (2001–2002) and IBenC study; Belgium participated only in the IBenC study. Although all the participating countries shared a trend towards demographic aging, considerable variations can be observed in population size between the countries and amount of home care provided in the countries. At the time of the IBenC study, the population size varied from 320,000 in Iceland to 80.5 million in Germany [12]. During the AdHOC study, Germany had the largest population, 82 million whereas the population of Iceland was only 286,000 [10]. The proportion of people older than 65 years, varies between the countries. In 2014, it was highest in Italy, with 31.6%, followed by Germany 31.2%, in Finland 27.7%, Belgium 26.4%, and Iceland 18.9% [12]. When the IBenC study was conducted, community care organizations were not-for-profit organizations in most countries, except in Germany, where 63% were for-profit organizations. In the other countries, the number of private for-profit organizations has been growing, which is perceived as a solution to the increasing demand for care. According to the IBenC data, only Iceland and Italy had hardly any private home care organizations [12]. In all participating countries, only a small proportion of the government expenditures on health care was on long-term home care, whereas the largest part was spent on acute care [12]. All participating countries emphasize the importance of integrated care and the importance of enabling older persons to stay at home for as long as possible [8, 12]. In Italian law, the importance of preventing older people’s social isolation is emphasised, but the importance of supporting informal caregivers is not mentioned [8].

Access to home care and funding for long-term care health services vary between the participating countries. In the IBenC study, care is mainly funded through public insurance, taxation, and client co-payments. In Germany and the Netherlands, it is provided primarily through obligatory public insurances, whereas in Belgium, funding depends on the type of care. Public insurance mainly funds care provided by nurses, whereas community taxation funds family care. In Finland, Iceland, and Italy, long-term care health services are mostly funded through national or municipal taxes [8]. Accessibility to home care varies and depends on the availability of care providers, reimbursement systems, and informal care expectations [15]. In Germany and Italy, access to home care is considerably lower than that in the Netherlands [15]. In countries where accessibility to home care is high, home care clients with relatively low dependency constitute the highest proportion of home care recipients, as observed in the Netherlands, Belgium, Finland, Italy, and Germany. Data for Iceland were not available [8, 15, 16].

The present study aimed to examine whether the characteristics of home care clients and the provision of home care in five European countries have undergone changes over the decade and to examine whether services offered met needs meaning that those in most need received most assistance. It was assumed that if the older persons had become frailer, had less functional ability, and required more complex treatments, they would have received more home care to remain living at home. It was also considered important that those who are deemed most in need of assistance receive the most care, more than those who are more self-reliant.

## Methods

In this descriptive study, data from two multi-national studies—the IBenC and AdHOC studies—were used. The AdHOC study results were obtained from published peer-reviewed articles [10, 17], and the data for comparison were obtained both from peer-reviewed article [18], and the IBenC database. In the comparison between these two studies and time periods only data from the countries that participated in both studies were used i.e., Finland, Germany, Iceland, Italy, and the Netherlands.

### Design and sample

The primary aim of the IBenC project was to identify best practices in home care taking into account both cost and quality [11, 18, 19]. Methods and sample descriptions of the IBenC study have been previously published [11, 18, 19]. In the participating countries, home care organizations in selected areas were invited to participate. Data heterogeneity was required for the development of the benchmark method. Organizations, preferably those using interRAI-HC, were selected based on the variety of their care practices rather than their representativeness. Data were collected simultaneously among three target groups: home care organizations, home care clients, and home care professionals. Only the data from the home care clients were used in the present study. The sample consisted of 2884 home care clients served by 38 home care organizations in the six participating European countries. Data collection followed a prospective longitudinal design with assessments at baseline and 6 and 12 months and was performed between January 2014 and August 2016. For this study, the baseline assessment data were used.

Care recipients are community-dwelling individuals receiving care from enrolled home care organizations. To be eligible for the study, the home care clients were required to be 65 years of age or older and be expected to remain in care for at least 6 months after initiating participation. The following individuals were excluded from the study: clients in the end stage of life, those who received care for a short period of time, those who were going to be institutionalized in the near future, and those with moderate cognitive impairment (Cognitive Performance Scale [CPS] score ≥ 3) without a known informal caregiver or legal representative. It should be emphasized that it was not the general population of older adults that was examined, rather a sample nearly representative of the ‘typical users’ of community home care services in the participating countries, because only limited number of home care organizations were included, and selection was based on variety in size, care practice, and location. Some selection bias may have been present in the samples from Italy and the Netherlands. In Italy, the baseline data were collected retrospectively for previous 6 months; thus, there was no dropout between the first two points of measurements, and the disability levels may have been overestimated. Cognitive impairment among elderly people receiving home care in the Netherlands was very low, probably because of the recruitment process in two of the three sites where one of the main reasons for refusal was cognitive impairment. Therefore, people with higher levels of cognitive decline were likely underrepresented in the sample from the Netherlands [15]. A limited number of organizations per country were selected based on the diversity in their location, size, care type, management, or payment form. The representativeness of the samples is thus uncertain, except that in Iceland, where the sample fully represents home care clients in the capital area.

The AdHOC study conducted in 2001–2002 focused on describing the aspects of users of home care services, specifically their health, functional status, and other aspects of living conditions [17]. The objective was to link the characteristics of community care recipients, the services they received, and the outcomes of care [10]. Inclusion and exclusion criteria for participating in the AdHOC study were the same as in the IBenC study—community dwelling individuals 65 years and older receiving home care. Further information on methods used in the AdHOC study have been published [10, 17].

### Procedure and measurement

All home care recipients participating in the two studies were assessed using the interRAI-HC, which is a comprehensive and structured geriatric instrument [20, 21]. The instrument is used in several countries in health care settings in routine care to support assessment and care planning for vulnerable patients’ groups as well as in research studies. The health status of an individual, as well as health problems, are extensively covered, providing an assessment of medical, psychological, social, and functional skills and care needs of dependent elderly living in the community [20–25].

The interRAI Home Care instrument has previously been shown to have inter-rater reliability across countries and settings [26]. The structure and predictive validity of the main scales and risk indicators that are embedded in the interRAI instruments have been tested extensively in multiple national and cross-national studies [27].

The instrument provides a range of data, both circumscribed information about issues such as hearing, vision, and activities of daily living and outcomes from scales that indicate levels of impairment. The scales are specially developed for the instrument and are part of it. In present study the functional status was evaluated using the Activities of Daily Living Hierarchy (ADLH) scale. This incremental scale highlights the loss of skills, both at an early and at a later stage. Fewer points are assigned for skills lost early, such as bathing, and more points are assigned for skills lost later, such as eating. The ADLH scale ranges from 0 (no impairment) to 6 (total dependence). For a score > 3 on the ADLH scale, extensive ADL support is required [28]. Cognitive status was assessed using the Cognitive Performance Scale (CPS), which ranges from 0 to 6. The CPS score indicates the ability to make decisions about everyday life activities and make oneself understood and memory impairment. A score > 3 indicates the presence of moderate to very severe impairment [29].

The care recipients’ characteristics such as gender, age, marital status, living alone, functional limitations, and cognitive function are documented. The total time provided by the home care nursing and social services in the previous 7 days was filled out, following instructions in the interRAI-HC manual. The total time for nursing and social services were added to determine the amount of home care time each client received.

### Statistical analysis

The results were analysed using descriptive and inferential statistics. Home care clients’ characteristics were reported, and comparisons were made between countries as well as between the two study periods. The analysis focused on cognitive and physical skills. The average hours of home care provision were compared between countries and between study periods.

Baseline data from the IBenC study were analysed to examine whether those who were most in need of care received more home care. The home care clients were divided into two groups according to physical and cognitive skills, based on scores on the ADLH scale and CPS, respectively. Those who scored 2 or lower were assigned to one group (low), and those who scored 3 or higher were assigned to another group (high). The cut-off point for needing considerable assistance was set at 3 for both scales [28, 29]. An individual with an ADLH scale score > 3 was considered in need of extensive ADL support, and an individual with a CPS score > 3 was considered to suffer from moderate-to-severe cognitive decline and in need of guidance [28, 29]. The duration of services provided by the home care (in min) in the 7 days before the evaluation were divided into three categories: 1–139 min (little), 140–419 min (moderate), and ≥ 420 min (substantial). The division is based on tradition and experience in home care. Home care for 139 min or less in the last 7 days, meant that clients receive only < 20 min of care per day. Limited assistance can be provided for 20 min per day, so clients who receive such aid are quite self-sufficient. When service is provided for up to 60 min a day, it means that the home care client needs considerable assistance, even twice a day, and probably receives assistance with dressing in the morning and going to bed at night. Clients who need assistance for more than 60 min a day may be significantly disabled and require severe assistance, even several times a day or from two care providers at a time. Cross-tabulation analyses were used to examine whether those most in need of assistance received home care for a longer duration than those in a better physical and cognitive condition. The chi-square test was used to test the significance of the difference between groups.

Correlation tests were performed to examine whether there was a correlation between high scores on the ADLH scale and CPS. It was also examined whether a high score on the ADLH scale or CPS affected the duration of home care received. Pearson’s correlation coefficient was used to indicate the strength and direction of the relationship. Analyses were performed using SPSS version 26.0.

## Results

Table 1 provides an overview of the sample in the two studies. The number of participants in the IBenC and AdHOC study was 2884 and 1808, respectively (from five countries). The average participant age was significantly (*p* < 0,05) lower in the AdHOC study (81.0 years) than in the IBenC study (83,0 years). The proportion of female participants was higher (p < 0,05) 73,0% in the AdHOC study but 66.9% in the IBenC study; in the latter study, the proportion was similar between countries but was by far the lowest in Italy (57.3%). On average, the majority of participants in both studies lived alone—54.3% in the AdHOC study and 59.4% in the IBenC study—but a great variance was observed between countries, from 16.4% in Italy to 80.9% in Finland in the IBenC study. The differences among the studies were found to be significant (*p* < 0.05).

**Table 1:** Characteristics of the home care clients in AdHOC and IBenC

	***Belgium***				***Finland***				***Germany***				***Iceland***				***Italy***				***the Netherlands***				***All***					
	*AcHOC*^*1)*^		*IBenC*		*AcHOC*^*2)*^		*IBenC*		*AcHOC*^*2)*^		*IBenC*		*AcHOC*^*2)*^		*IBenC*		*AcHOC*^*2)*^		*IBenC*		*AcHOC*^*2)*^		*IBenC*		*AcHOC*		*IBenC*		*IBenC*^*4)*^	
**Study sample** - (n)				(525)		(187)		(456)		(607)		(493)		(405)		(420)		(412)		(499)		(197)		(491)		(1808)		(2884)		(2359)
**Female - %** (n)			67.0	(352)	82.2	(154)	68.6	(313)	74.7	(453)	71.2	(351)	74.1	(300)	69.5	(292)	63.1	(260)	57.3	(286)	77.3	(152)	68.4	(336)	73.0	(1319)	66.9	(1930)	66.9	(1578)
**Living alone -** % (n)			48.0	(252)	83.8	(157)	80.9	(369)	62	(375)	73	(359)	68.2	(276)	61.0	(256)	12.8	(53)	16.4	(82)	61.6	(121)	68.8	(338)	54.3	(982)	57.4	(1656)	59.4	(1404)
**Age,** years - mean (SD)			82.4	(6.7)	81.2	(7.7)	82.7	(7.0)	81.3	(7.9)	84.2	(7.6)	81.4	(7.6)	83.7	(7.0)	80.2	(8.0)	81.8	(7.9)	80.4	(6.7)	82.5	(7.1)	81.0	7.6	83.0	(7.3)	83.0	(7.4)
**CPS score** - mean (SD)			1.4	(1.6)	0.7	(1.0)	1.3	(1.2)	1.4	(1.8)	1.6	(1.7)	0.6	(1.0)	1.1	(1.2)	2.0	(2.0)	2.4	(2.1)	1.0	(1.1)	0.6	(0.9)	1.1	(1.2)	1.4	(1.6)	1.4	(1.6)
**ADLH score** - mean (SD)			3.2	(1.2)	0.2	(0.9)	0.8	(1.3)	1.6	(1.8)	2.2	(1.7)	0.2	(0.7)	0.6	(1.1)	2.8	(2.0)	3.8	(1.7)	0.2	(0.8)	0.5	(1.1)	1.0	(0.7)	1.9	(2.0)	1.6	(1.9)
**Hours of formal care** - mean (SD)^*3)*^			8.5	(7.8)	2.2	(1.0)	5.1	(5.2)	2.7	(0.8)	7.5	(6.9)	2.2	(0.9)	3.6	(3.8)	1.3	(0.7)	1.0	(2.6)	2.6	(0.9)	4.6	(4.7)	2.2	(0.8)	5.1	(6.1)	4.4	(4.6)

^1)^ Belgium did not participate in AdHOC

^2)^ Data for AdHOC from [8] in the reference list

^3)^ Data for IBenC from [11] in the reference list

^4)^ IBenC without Belgium

**Fig. 1:**
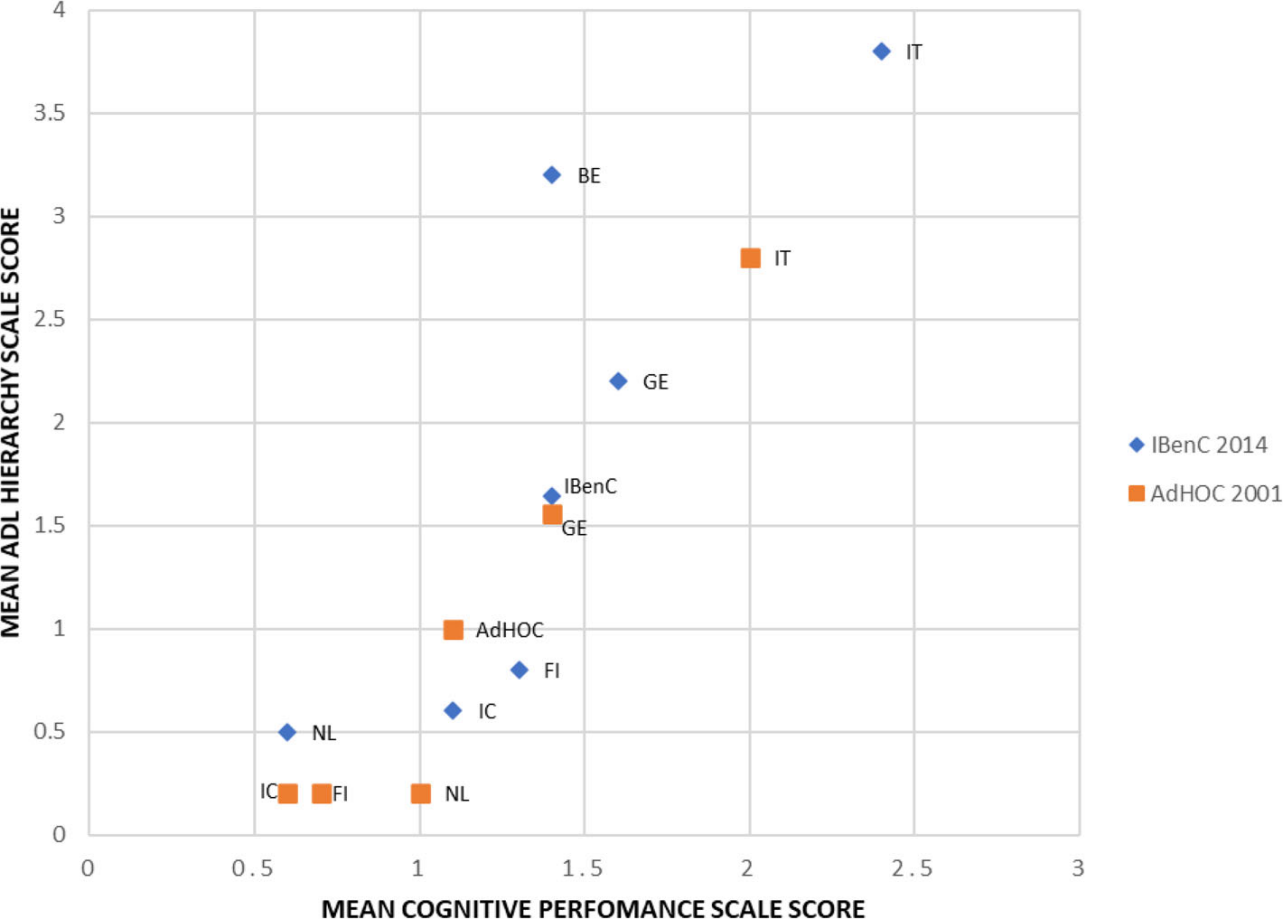
Relationship between mean Cognitive Performance Scale score and mean ADL Hierarchy scale score by country in the AdHOC □ and IBenC ◊ samples. BE = Belgium, FI=Finland, GE = Germany, IC=Iceland, IT = Italy, NL = the Netherlands. AdHOC = mean values in the AdHOC study and IBenC = mean values in the IBenC study. Mean score in AdHOC and IBenC is without Belgium. Chi-square test *p* < 0,05

Regarding home care recipients’ physical and cognitive skills, the mean CPS and ADLH scale scores were significantly (p < 0,05) higher in 2014 (IBenC) than in 2001 (AdHOC) (Fig. 1). The CPS score was also significantly (*p* < 0,05) higher in 2014 in all countries except the Netherlands, where it was lower; it was also the lowest CPS score (0.6) reported in 2014, whereas the home care clients in Italy had the highest CPS score (2.4). The mean score on the ADLH scale was significantly (p < 0,05) higher in the IBenC study (1.6) than that in the AdHOC study (1.0). In the IBenC study, home care clients in Italy scored the highest both on the physical and cognitive scales (Fig. 1), indicating that they had the highest care needs, whereas home care clients in Iceland and the Netherlands showed the lowest levels of cognitive and functional decline. These findings were consistent with the findings from the AdHOC study.

**Fig. 2:**
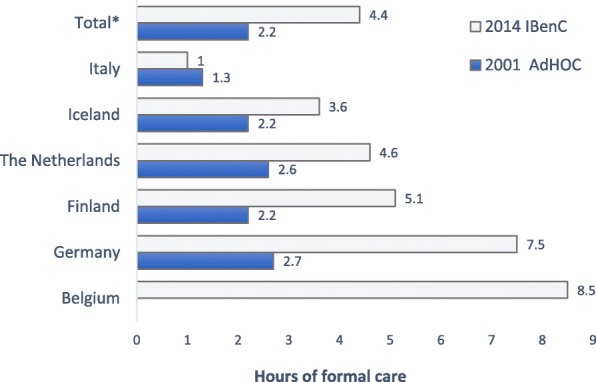
Hours of home care the last 7 days before evaluation. Difference between countries and studies are significant *p* < 0,05. * Total for Italy, Iceland, the Netherlands, Finland, and Germany. Belgium didn’t participate in AdHOC. Chi-square test *p* < 0,05

The duration of home care nursing and social services provided in the previous 7 days was significantly higher (*p* < 0,05) in the IBenC study (4.4 h) than in the AdHOC study (2.2 h). The duration of home care provided was the lowest in Italy (1.0 h) and the highest in Belgium (8.5 h) (Fig. 2).

**Fig. 3:**
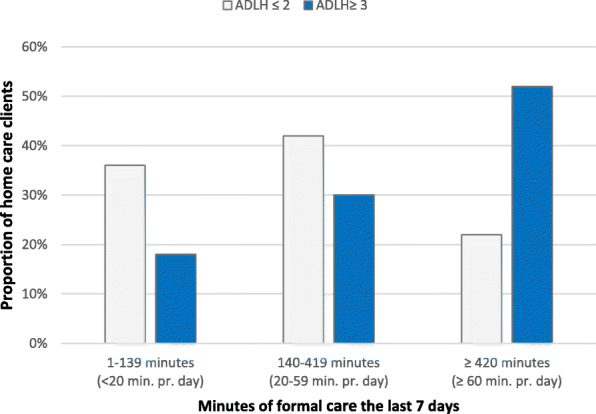
Relationship between score on ADLH scale and the number of minutes of home care received for the countries together in the IBenC study, the last 7 days before evaluation. Chi-square test *p* < 0,05

**Fig. 4:**
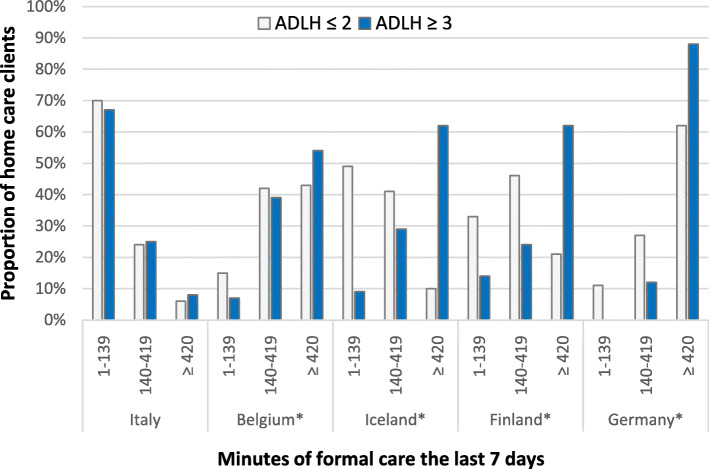
Relationship between score on ADLH scale and the number of minutes of home care received for each country in the IBenC study, the last 7 days before evaluation. *significant Chi-square test *p* < 0,05

A cross-tabulation analysis of the score on the ADLH scale and duration of home care received (Fig. 3) found a significant relationship (*p* < 0.05) between physical impairment and getting the most assistance from home care. Results indicated that more than half of those who had severe physical impairment received substantial home care, whereas 30 and 18% received moderate and little assistance, respectively. Over 20% of those who did not have severe physical impairment received substantial help. Cross-tabulation analyses performed for each country showed a significant relationship (p < 0.05) between severe physical impairment and substantial home care received in Iceland, Finland, Belgium, and Germany (Fig. 4). In Germany, 88 and 12% of those who scored high on the ADLH scale received substantial and moderate assistance, respectively. In Iceland and Finland, 62% of those who scored high received substantial care, but 24 and 29% received moderate assistance, respectively, and 9 and 14% received little assistance, respectively.

**Fig. 5:**
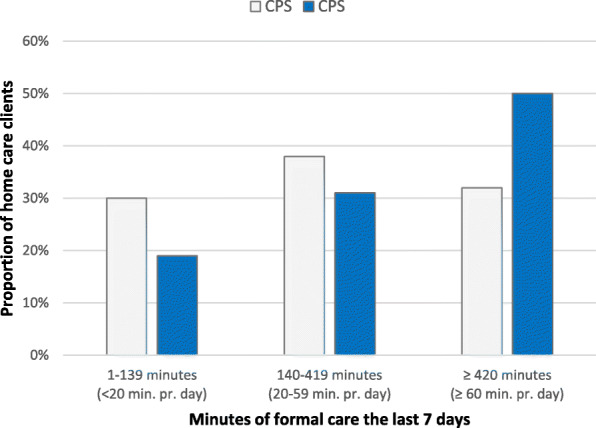
Relationship between score on CPS scale and the number of minutes of home care received for the countries together in the IBenC study, the last 7 days before evaluation. Chi-square test *p* < 0,05

**Fig. 6:**
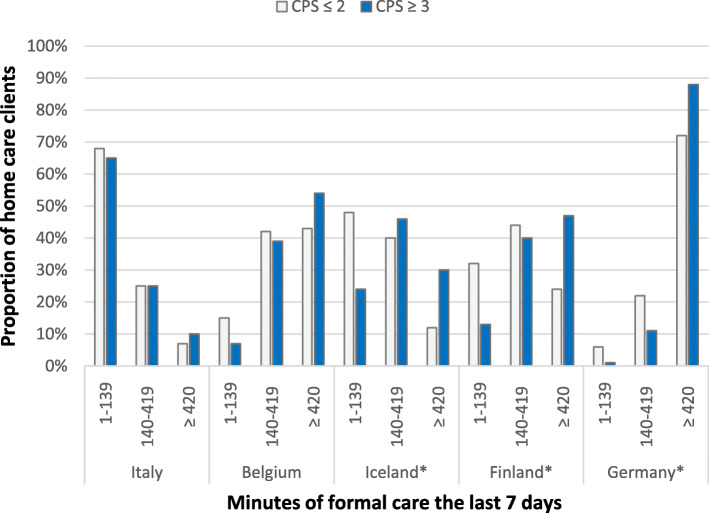
Relationship between score on CPS scale and the number of minutes of home care received for each country in the IBenC study. *significant Chi-square test *p* < 0,05

The duration of home care received was significantly higher for home care clients with moderate-to-severe cognitive impairment than for those who scored lower on the CPS (*p* < 0.05), as seen in Fig. 5. Cross-tabulation analysis performed for each country individually (Fig. 6) indicated a significant positive relationship (*p* < 0.05) between the severity of cognitive impairment and duration of assistance from home care in Iceland, Finland, and Germany. Home care clients in Germany received more assistance than home care clients in the other countries regardless of whether they had mild or severe cognitive impairment. In Iceland, 30, 46, and 24% of home care clients with severe cognitive impairment received substantial, moderate, and little home care, respectively. In Italy, only 10% of those with severe cognitive decline received substantial home care. The data on the duration of home care received by home care clients in the Netherlands were not analysed as the number of responses was very low.

A significant, moderate positive correlation (r = 0.447, *p* > 0.05) was observed between high scores on the ADLH scale and CPS. There was also a significant, weak positive correlation (r = 0.349, *p* > 0.05) between a high score on the ADLH scale and receiving substantial home care, but a very weak correlation (r = 0.154, *p* > 0.05) was observed between the CPS score and duration of home care received.

## Discussion

The proportion of older people in the society has risen, and governmental policies worldwide have been clear in supporting people with care needs to continue living at home. It is important for governments and home care organizations to be aware of the home care clients’ characteristics and skills when they organize home care services and estimate the costs.

The findings of this study indicate that in the 10 years between the AdHOC and IBenC studies, the physical and cognitive skills of home care clients in several European countries have deteriorated, indicating that older people can remain living in their homes for longer if increased home care. Similar findings were reported in Sweden [30] and in Ontario in Canada, where a clear deterioration was observed in home care clients’ physical and cognitive abilities between 2003 and 2014 [31].

As stated earlier, the government policy has been to enable people with complex needs to live in their own homes for an extended period. Many countries have followed this policy and changed the emphasis and work processes as well as providing additional and more diverse service resources. Health authorities in the province of Ontario in Canada have emphasized discharging people home from acute care settings before admitting them to long-term care institutions. Increased home care resources in Ontario enable home care clients to live at home longer despite more complex health problems [31]. Another example is Iceland, where the government has been working on integrating health and social services and increased cooperation between emergency services and primary care [32, 33]. Various steps have also been taken to improve collaboration and information sharing between these two services, for example, development of clinical pathways for the treatment of heart failure [34, 35] and opening electronic access to hospital health records for home care nurses. Home care nurses indicate that these measures have changed the working conditions in home care nursing so that frail older people with more complex health problems can live at home.

In this study, half of those who most needed care, that is, those who scored high on the CPS and the ADLH scale received most minutes of assistance. One-third and one-fifth of those who most needed care received moderate and little service, respectively; thus, there was a group of clients who did not receive the needed home care, as indicated in Figs. 4 and 5. There was a moderate correlation between the score on the ADLH scale and duration of home care received. Therefore, it can be said that the services are prioritised, to a certain extent, for those who are most in need.

A group of home care clients in the study did not have considerable needs but received substantial assistance. Although there was a moderate correlation between high scores on the ADLH scale and CPS, there were some home care clients who were in good physical condition but showed severe cognitive impairment and therefore required substantial assistance. It can be assumed that these home care clients partly explain why one-fifth of those who scored low on the ADLH scale received substantial assistance (Fig. 4) and why just over a third of those who scored low on the CPS received substantial assistance (Fig. 5).

Although home care clients receive moderate-to-substantial service in minutes, it cannot be generalized from these data whether the need for service is fully met. Other studies have shown that home care provided does not always meet the needs. In a study conducted in Cyprus, home care clients reported that they were satisfied with the home care nursing; however, they also wished for more home visits, both from nursing and social care. However, some of them said that they did not receive care and advice regarding their psychological needs; thus, it appears that certain care needs are not met even though assessment for assistance has been conducted [36]. In another study conducted in Norway, the defined care needs of home care clients, as identified by an expert panel, were fulfilled in more than 60% of cases (e.g. clients needing skin and wound care, monitoring of blood glucose in clients with diabetes, and supporting the food intake of those with eating difficulties). Other defined needs were only fulfilled in less than 10% of cases. The authors emphasized that home nursing resources should be more flexible and more proactive to preserve functional status and prevent avoidable hospitalizations [2].

Studies have shown that provision of formal assistance is not equitable. The Assessing Needs for Care in European Nations (ANCIEN) project focused on the future of long-term care (LTC) for the elderly in Europe and addressed questions related to how need, demand, supply, and use of LTC will develop [37]. The project also examined the performance of different systems of LTC. Two types of equity were analysed: equity of revenue raising and equity of resource allocation, further divided into equity of access and equity in levels and mix of services relative to needs. In Germany and Belgium, individuals with the same level of needs were able to access the home care system in the same manner and obtain the same care. However, individuals with higher levels of care needs did not necessarily receive more care although they had more access to the home care system. The ANCIEN project concluded that, in Finland and Italy, there was no system to ensure that people with the same level of need could access the home care system equally [12, 37, 38]; the same can be said about the findings of this study—although home care clients had high scores on the ADLH scale and CPS, not everyone received substantial home care.

Another study that used the data from the IBenC study reported substantial differences between provided and expected formal care both within and across countries that the case-mix differences of the recipients could not explain. It can be concluded that the provision of equal home care services based on need may be challenging [39]. Variation across countries can be expected as allocation criteria and availability of formal or informal resources differ. Cultural expectations and legal requirements related to informal care involvement produce a varied balance between formal and informal care [39].

The results presented here provide an opportunity to map the changes in home care clients’ characteristics and home care in several European countries. There is an indication that the government policy has been successful to some degree, as people with higher dependency levels live independently for longer periods. It also appears that governments in the countries included in the studies have adhered to their policy by increasing home care to home care clients, except in Italy, where it has slightly decreased. The findings suggest that home care clients’ skills vary among countries, and so does the duration of home care provided by the formal system.

In Iceland, the sample was fully representative of home care clients in the capital area, where over 60% of the country’s population lives. The average scores on the ADLH scale and CPS were low but have risen over the 10 years between the two studies. Compared to the other participating countries in the IBenC study, the home care is more limited in Iceland; however, it has improved in the last decade. In those countries where more home care is provided, home care clients have higher physical and cognitive impairments. It appears that increased home care enables people with reduced physical and cognitive abilities to stay longer in their own homes. If the government in Iceland intends to achieve its goal of elderly with complex needs living longer in their own homes, one of the factors required to reach that goal is increasing home care. Italy is an exception because home care is limited and access is not directly related to need because families have an enormous moral and legal responsibility to care for their elderly family members [18].

This study focuses on the relationship between home care clients’ cognitive and physical skills and hours of home care provided, and findings indicate that those who need help due to physical and cognitive impairment do receive assistance but not whether such assistance meet their needs. The fact that the analysis only focused on the relationship between clients’ physical and cognitive impairment and home care hours provided is a limitation in this study. Other factors, such as diseases, intensity and presence of pain, depression, and assistance from family and friends (i.e. informal care), could also have influenced the time of home care provided to clients. In future research, the interplay of these factors needs to be examined, and interRAI assessments afford many opportunities to do so. The type of home care was not distinguished in this study, but it could be informative to identify the assistance provided by nurses, health care assistants, and domestic care workers. However, the major strength of the present study was the use of the internationally validated and reliable interRAI-HC with trained assessors.

## Conclusions

It is concluded that the government policy of supporting older people to live longer in their own homes may have been successful to a certain extent. The physical and cognitive skills of home care clients living at home declined during the past decade. To meet the growing need for assistance, governments have increased their provision of home care to home care clients. In this study, the duration of assistance provided could be determined, but the kind of assistance provided could not be identified. Half of the home care clients who most needed assistance received substantial home care. It is important to examine why the other half only received moderate or little assistance; this factor needs to be investigated more closely, and approaches to provide the required service need to be identified. In future studies, it is also important to further investigate the allocation of services – whether those in most need get the assistance they need – and examine other factors not included in this study. As the number of older adults increases, it is expected that more elderly people will need assistance, and as there will be fewer working hands, it is important services are allotted fairly or provided as needed.

The home care provided must meet individual care needs, be flexible and pro-active to prevent further health decline, and maintain skills for continued residence in private homes.

## Data Availability

The datasets used and/or analyzed during the current study are available from the corresponding author on reasonable request and with permission of the IBenC consortium.

## Abbreviations

AdHOC: AgeD in the Home Care
IBenC: Identifying best practices for care-dependent elderly by Benchmarking Costs and outcomes of Community Care
interRAI-HC: interResident Assessment Instrument for home care
MDS: Minimum data set
CAPs: Client assessment protocols
RUGs: Resource utilization groups
CPS: Cognitive Performance Scale
ADLH: Activities of Daily Living Hierarchy scale
ANCIEN project: Assessing Needs for Care in European Nations
LTC: Long Term Care
